# Gait changes precede overt arthritis and strongly correlate with symptoms and histopathological events in pristane-induced arthritis

**DOI:** 10.1186/ar2950

**Published:** 2010-03-11

**Authors:** Markus H Hoffmann, Rudolf Hopf, Birgit Niederreiter, Heinz Redl, Josef S Smolen, Günter Steiner

**Affiliations:** 1 Division of Rheumatology, Internal Medicine III, Medical University of Vienna, Waehringer Guertel 18-20, A-1090 Vienna, Austria; 2 Ludwig Boltzmann Institute for Experimental and Clinical Traumatology in the AUVA Research center for Traumatology and Austrian Cluster for Tissue Regeneration, Donaueschingenstrasse 13, A-1200 Vienna, Austria

## Abstract

**Introduction:**

Pristane-induced arthritis (PIA) in the rat has been described as an animal model of inflammatory arthritis which exhibits features similar to rheumatoid arthritis in humans, such as a chronic, destructive, and symmetrical involvement of peripheral joints. However, so far little is known about the earliest inflammatory events and their influence on locomotor behaviour during the course of PIA. To investigate this issue a detailed analysis of the pathologic changes occurring during the prodromal and early stages of PIA was performed.

**Methods:**

Arthritis was induced in DA.rats by injection of 150 μl 2,6,10,4-tetramethyl-pentadecane (pristane) at the base of the tail and changes in locomotor behaviour of the affected paws were monitored using the CatWalk quantitative gait analysis system. The pathologic events occurring in the joints of pristane-injected animals were studied before onset, at onset, and during acute phase of arthritis by histological methods.

**Results:**

Gait analysis revealed that changes in locomotion such as reduced paw print areas and stance phase time are already apparent before the onset of clinically discernible arthritis symptoms (erythema, paw swelling) and correlate with PIA scores. In agreement with these findings, inflammatory tenosynovitis could be observed by histology already before the onset of erythema and swelling of the respective paws. In the most heavily affected rats also irregularities in step sequence patterns occurred A kinetic analysis of clinical and histological findings demonstrated that gait changes precede the pathological changes occurring during the acute phase of pristane-induced arthritis.

**Conclusions:**

Gait analysis allows for pinpointing the initial inflammatory changes in experimental arthritis models such as pristane-induced arthritis. Analysis of early clinically relevant symptoms in arthritis models may facilitate the search for novel therapeutics to interfere with pain, inflammation and joint destruction in patients suffering from inflammatory arthritis.

## Introduction

Arthritis is a clinical entity characterized by joint swelling, stiffness and spontaneous or motion-related pain. Impairment of physical function, that is, disability, is the major complication of arthritis and the degree of its reversibility depends on both our potential to interfere with the active disease process as well as with joint damage, given that the latter is currently not reversible [1].

Our current knowledge on the pathogenesis of chronic inflammatory joint diseases such as rheumatoid arthritis (RA) is still limited. While evidently there are a variety of important genetic factors involved [2] and a large proportion of patients develops autoantibodies consistent with the involvement of an autoimmune response, neither the trigger(s) of disease are known nor are the pathways leading to chronicity fully understood. Therefore, animal models of arthritis are very useful in dissecting these pathways, although each of them may depict only a facet of RA or other forms of inflammatory joint disease. To a large extent these models rely on the assessment of disease activity, which is hampered by the inability to obtain subjective symptomatological information and, therefore, is at best semi-quantitative in nature. Also, since physical function, the most important endpoint in patients with arthritis, is usually assessed by questionnaires [3], disability cannot be evaluated in a similar way in experimental animal models. Therefore, tools which could address disability to allow comparison between different models or allow us to improve our understanding of the effects of therapeutic interventions would be highly valuable.

In the present study we have characterized the effects of varying degrees of severity of pristane-induced arthritis (PIA) in rats on physical function using the CatWalk method. Arthritis can be induced in susceptible rat strains such as Dark Agouti (DA) or Lewis by a single intradermal injection of the chemically inert mineral oil pristane. The induced disease closely mimics human RA as it fulfils many of the clinical criteria of RA including a symmetrical involvement of peripheral joints, the presence of rheumatoid factor and anti-RA33 antibodies [4], destruction of cartilage and bone, and a chronic disease course [5]. Arthritis in the DA rat develops suddenly and dramatically around two weeks after pristane-injection. An episode of severe and destructive arthritis in the peripheral joints follows and gradually subsides several weeks later. Subsequently, a chronic relapsing disease develops in most of the animals which can reach almost as high a severity as during the first arthritic episode and does not subside [6].

The CatWalk-system was originally developed for studying rats with spinal cord injuries [7], and is a video-based system for automated gait analysis that enables to measure a number of variables pertaining to gait pattern and weight-bearing. CatWalk has recently been used to assess gait changes caused by carrageenan-induced arthritis [8,9] and adjuvant-induced arthritis [10]. Similar systems employing a treadmill instead of a flat surface for walking were applied for characterizing adjuvant arthritis in the rat and collagen-induced arthritis in the mouse [11,12]. Furthermore, thermal imaging of paws can provide reliable information about the degree of joint inflammation in murine and rat models of arthritis [13,14].

In the present study arthritic rats were followed from the preclinical and early stages of arthritis, over the maximum severity to the remission after the acute phase of PIA. It will be shown that severe arthritis dramatically affects the walking pattern and thus physical function in this experimental model. Importantly, since gait changes are detectable already before the onset of clinically visible symptoms of arthritis such as erythema and swelling, automated locomotor analysis may be of particular interest to define the earliest processes involved in a very severe and debilitating disease such as PIA.

## Materials and methods

### Animals

Dark Agouti (DA) rats (originating from Zentralinstitut für Versuchstierzucht, Hannover, Germany) were bred and maintained under Specific Pathogen Free (SPF) conditions in the animal facility of the Institute of Biomedical research, Medical University of Vienna, Austria. Rats were housed in groups of two to three in plastic cages with 12 h light/dark-cycles. Standard rodent chow and water was provided ad libitum. Experiments were performed on age- and sex-matched animals. All experiments were approved by the local ethical committee.

### Induction and evaluation of pristane-induced arthritis

Arthritis was induced in rats at the age of 8 to 12 wk by a single intradermal injection at the base of the tail with 150 μl of pristane oil (2,6,10,4-tetramethylpentadecane; Sigma-Aldrich, St. Louis, USA). Control rats received intradermal injection of sterile phosphate-buffered saline (PBS; PAA Laboratories, Pasching, Austria). Rats were randomly allocated to pristane- or PBS-groups. Arthritis development was monitored by a blinded observer in all four limbs using a semiquantitative scoring system as previously described [15]. Briefly, one point was given for each swollen or red finger/toe (erythema or swelling of distal or proximal interphalangeal joint or both), one point was given for each swollen or red metacarpophalangeal/meta-tarsopahalangeal joint, and one to five points were given for a swollen wrist/ankle, depending on severity (the maximum score per limb and rat was 15 and 60, respectively). The rats were scored at days 0, 6, 9, 11, 12, 14, 16, 19, 24, 29 and 35 after injection.

### Assessment of pain- and arthritis-induced gait changes with the CatWalk method

The CatWalk system (Noldus Information Technology, Wageningen, The Netherlands) has been described in detail elsewhere [7]. In short, it is a video-based system for automated gait analysis using an enclosed walkway with a glass floor where light enters along one long edge. At the placement of a paw, the light projected into the glass floor is scattered and produces an illuminated print. Using this method it is possible to measure a number of gait and weight bearing-variables. To avoid possible misinterpretation, the analysis was performed using a threshold value for skin-floor contact of 44 arbitrary units (a.u, possible range 0 to 255), that is, all pixels brighter than 44 were used.

In this study the following variables were analysed:

*1. Print area*:

The total floor area contacted by the paw during stance phase, that is, the area that would be blackened if the animal's paw had been painted with ink.

*2. Duration of stance phase*:

Since the absolute duration of stance or swing phase depends on the animal's walking speed, these variables are transformed to a fraction of total step duration according to the following formula: fraction stance phase = stance phase duration/(stance phase duration + swing phase duration), where the stance phase duration is the time of contact of one paw with the floor during a single stepcycle and the swing phase duration is the time of non-contact with the floor during the stepcycle.

*3. Regularity index*:

Interlimb coordination is complete if only normal step sequence patterns occur during uninterrupted locomotion (that is, when the animal places the four paws one after another). The regularity index grades the degree of interlimb coordination as follows: RI = (NSSP × 4/PP) × 100 (%) wherein *NSSP *represents the number of Normal Step Sequence Patterns and PP the total number of Paw Placements. In healthy animals this value is 100%.

Testing was performed between 09:00 and 16:00 h on age-matched DA. 1F rats. All animals were first allowed to habituate in and to cross the walkway before recording of runs. Testing was conducted by the same person. At each test occasion the animals were allowed to walk until a satisfactory recording was acquired. A satisfactory recording was defined as uninterrupted locomotion for a sufficiently long distance of the walkway to get at least three to four paw-placements by each non-arthritic paw. If the animal stopped to explore the environment by rearing, turned around to walk in the opposite direction or if it refused to walk far enough, the recording was repeated. A >5% reduction of print area or shortening of stance phase time versus pre-operative values was considered a significant change in locomotor behaviour.

### Histological examinations

Paws from rats 11 and 35 days after injection of pristane or PBS were collected and decalcified with ethylenediaminetetraacetic acid (EDTA). Serial paraffin-embedded tissue sections of hind and front paws were analysed by H&E staining, by tartrate-resistant acid phosphatase (TRAP) staining (leukocyte acid phosphatase kit; Sigma-Aldrich) for the identification of osteoclasts, and by toluidine blue staining for visualizing cartilage. Areas of synovial inflammation, erosions of cartilage and bone, and number of osteoclasts were determined by histomorphometry using an Axioskop 2 microscope (Zeiss, Oberkochen, Germany) and the OsteoMeasure analysis software (Osteometrics, Decatur, GA, USA).

### Statistical analysis

For each walk of an animal, the mean was calculated per paw for print area, stance and swing duration. Regularity index was also obtained. Mean values for all variables were then calculated for pristane- and PBS-injected rats. Comparisons between gait variables in control and pristane groups were made by ANOVA tests. Where significant effects were found, subsequent comparisons were performed using Dunnett's test. Comparisons with pre-morbid values within groups (gait variables, arthritis scores, and histological assessments) were statistically tested using paired t-tests. To analyse correlations between arthritis score area under the curve (AUC) and area under the curve of gait variables, Pearson's correlation coefficients were calculated. All data are expressed as mean ± Standard error of the mean (SEM).

## Results

### Analysis of pristane-induced gait changes by CatWalk

**Figure 1:**
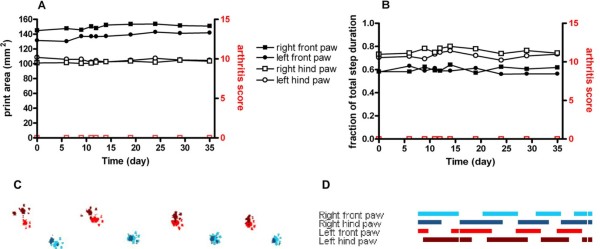
**Gait analysis by CatWalk in a healthy rat**. Time course of the print area **(A) **and the duration of stance phase measured as the fraction of total step duration **(B) **of all four paws in a 10-week-old female control rat walking across the CatWalk walkway. Front paws are shown with filled symbols, hind paws with open symbols. Extremities of the right side are indicated as squares, of the left side as circles. Gait variables are depicted in black, arthritis scores in red. **(C) **Picture of CatWalk footprints on Day 24 after injection of PBS. Prints of the left paws of the rat are shown in red, and prints of the right paws are shown in blue. Prints of front paws are depicted in lighter colour, whereas prints of the hind paws are darker. **(D) **Picture of the CatWalk gait diagram 24 days after PBS-injection. Indicated are the paw-floor contacts for each of the paws over time. The length of each bar represents the duration of the stance phase for that particular paw. The space between bars represents the duration of the swing phase. No conspicuous changes in gait variables were detected.

PBS-injected (control) rats did not develop arthritis and print area and stance phase remained constant over the analysed period of time (Figure 1). In these animals during uninterrupted locomotion only complete step cycles were performed. Interestingly, in the rats tested there was a significant difference in print area between front and hind paws, with front paws covering a larger area (149.30 ± 0.66 mm^2 ^vs 96.67 ± 0.73 mm^2^, *P *< 0.0001, Figure 1A, C). Furthermore, the fraction of the stance phase duration relative to the total step duration was significantly longer for hind legs compared to front legs (0.747 ± 0.004 vs 0.593 ± 0.003, *P *< 0.0001, Figure 1B, D). In contrast, no significant differences were observed at any time over the test period between the left and right sides for print area and stance phase time.

**Figure 2:**
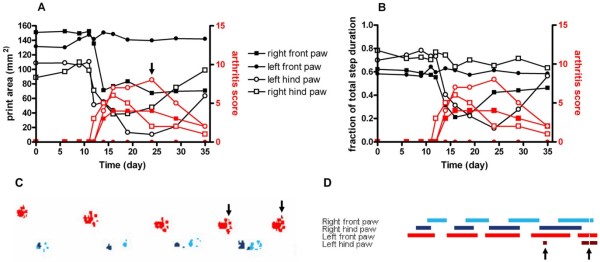
**Gait analysis by CatWalk in an arthritic rat**. Time course of the print area **(A) **and the duration of stance phase measured as the fraction of total step duration **(B) **of all four paws in a nine-week-old female pristane-treated rat walking across the CatWalk walkway. Front paws are shown with filled symbols, hind paws with open symbols. The left side is shown with circles, the right side with squares. Gait parameters are depicted in black, arthritis score in red. **(C) **Picture of CatWalk footprints on Day 24 after injection of 150 μl pristane oil. Prints of the left paws of the rat are shown in red, and prints of the right paws are shown in blue. Prints of front paws are depicted in lighter colour, whereas prints of the hind paws are darker. **(D) **Picture of the CatWalk gait diagram 24 days after pristane-injection. Indicated are the paw-floor contacts for each of the paws over time. The length of each bar represents the duration of the stance phase for that particular paw. The space between bars represents the duration of the swing phase. Decreases in print area as well as in the stance phase in the arthritic paws are observed (arrows).

Figure 2 shows the influence of arthritis on print area and stance phase in the individual paws of a pristane-injected rat. Arthritis started at Day 11 after pristane-injection in the right hind paw and at Day 12 in two other paws; its occurrence was mirrored by a dramatic decrease in print area of the affected paws (Figure 2A). At the peak of the acute phase on Day 24 the print area of the most severely affected left hind paw was reduced to less than 10% of its pre-morbid value (Figure 2A, C). Also the stance phase duration was heavily affected by arthritis, although only two of three arthritic paws exhibited a shortening of the stance-phase (Figure 2B). This can be interpreted by the rat's compromise between a minimization of pain and the maintenance of functional walking. The illustrations of the CatWalk foot prints reveal that on Day 24 the most heavily affected paw is not used during some of the step cycles (Figure 2C, D). With the gradual remission of arthritis from Week 4, gait parameters were partially restored to their pre-morbidity values (Figure 2A, B).

### Gait changes mirror the clinical course of PIA and reflect histopathological events in the joints

Untreated rats that walk across the walkway perform a regular pattern of locomotion, placing the paws one after another. The time normal control rats used for crossing a 90 cm distance was about 1.52 ± 0.039 s (velocity about 59.21 cm/s or 2.13 km/h), whereas rats with arthritis crossed the walkway more slowly, with mean velocity significantly decreasing to about 30.96 cm/s (1.11 km/h) (*P *< 0.0001).

**Figure 3:**
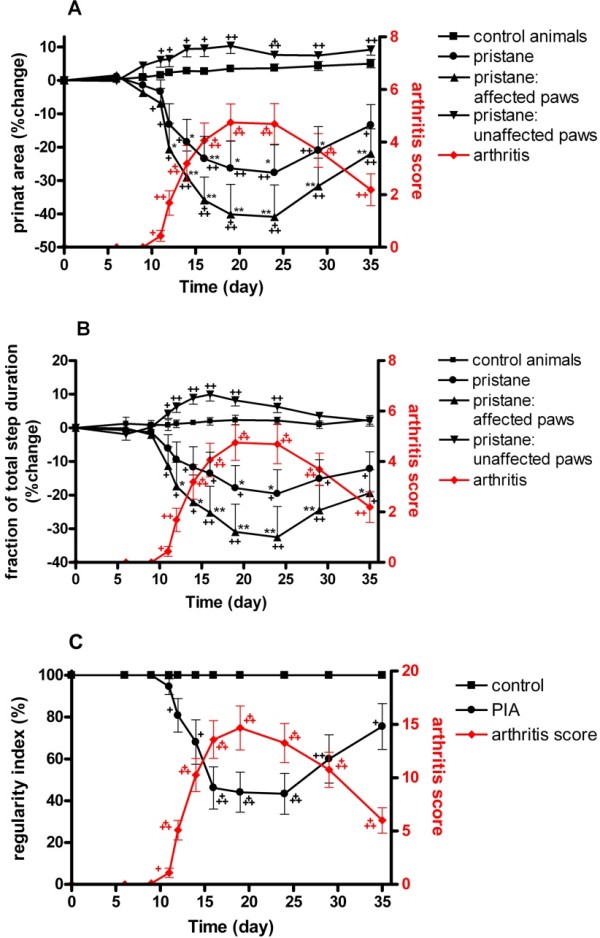
**Longitudinal gait analysis of pristane-induced arthritis**. Time course of the print area **(A) **and the stance phase-fraction of total step duration **(B) **of paws of pristane-injected versus control rats. Mean values obtained for the gait variables of affected (n = 16) and unaffected (n = 8) paws are shown in black. Respective values of all paws of pristane-injected animals (n = 24) are also depicted for comparison. Arthritis scores of affected paws are depicted in red. Mean values ± SEM of paws were calculated and subjected to ANOVA with Dunnett's post hoc test to compare paws of pristane-injected animals with paws of control animals. * *P *< 0.05, ** *P *< 0.01. For longitudinal analysis within each group, locomotion and arthritis score was recorded prior to injection and was used as the baseline reading (Day 0). Comparisons with later time points were performed using paired t-tests. + *P *< 0.05, ++ *P *< 0.01, +++ *P *< 0.001. One representative out of two experiments is shown **(C) **Time course of the regularity index measured by the percentage of normal step sequence patterns in naive control rats (n = 8) and rats injected with pristane (n = 12). Overall arthritis scores of affected animals are depicted in red. The curves show mean values ± SEM. Differences to pre-morbid values were calculated using paired t-tests.

The print areas of arthritic paws in pristane-injected rats were significantly smaller than print areas of unaffected paws and paws from PBS-injected control animals from Day 12 until the end of the observation time at Day 35, although they gradually came closer to control values towards the end of the observation (Figure 3A). Equally, the time of contact with the floor was significantly decreased in arthritic paws from Days 12 to 35, but was gradually approaching control values after Day 24 (Figure 3B). Interestingly, both the print area and the stance phase of non-arthritic paws from pristane-injected animals were markedly increased when compared with paws from control animals, suggesting a transposition of weight bearing from the arthritic to the non-arthritic limb. A second independent experiment achieved comparable results, showing significant reduction of print area and stance phase from Day 11 in affected paws with a concomitant increase in non-affected paws from pristane-injected animals (data not shown).

Effects on interlimb coordination by PIA are measured by the CatWalk method as a decrease in % Regularity Index (RI) (Figure 3C). Healthy, fully coordinated animals show only normal step sequences (RI = 100%), but in arthritic rats that exhibited loss of paw placements of affected paws, the RI decreased in parallel with the increasing severity of arthritis. With the remission of inflammation in the paw, the affected paws could be used normally again and RI returned towards normal values.

**Figure 4:**
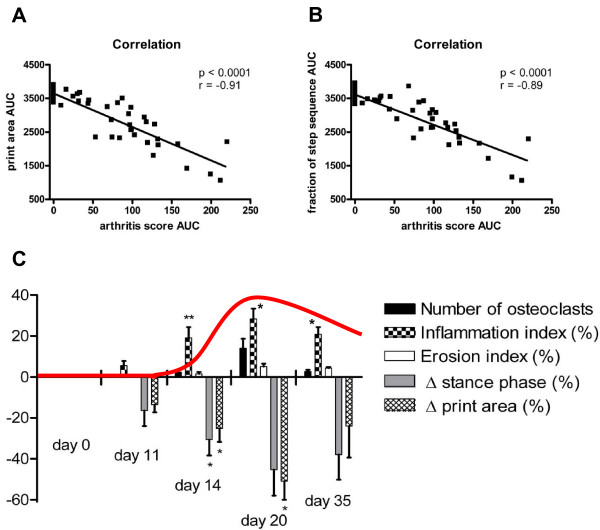
**Clinical variables and histopathological changes of PIA correlate with gait changes measured by CatWalk**. X-Y scatter plots of correlations between arthritis score AUC and print area AUC **(A) **and stance-phase fraction of total step duration AUC **(B) **of individual paws (n = 80) from pristane-injected and control animals are shown. Pearson coefficients (r) and *P*-values are depicted within the graph. (C) Rats were monitored by CatWalk and sacrificed at characteristic time points (Day 0 = naïve animals, Day 11 = preclinical phase, Day 14 = onset, Day 20 = highest severity, Day 35 = begin of remission). The areas of inflammation, erosion, and the numbers of osteoclasts were determined histomorphometrically and adjusted to the total area of tissue analysed. The course of clinically apparent PIA is shown as an overlay in red. Each bar represents mean ± SEM from three arthritic paws.

When comparing clinical arthritis scores with changes in gait, we found highly significant correlations (r = -089 and r = -0.91, respectively, *P *< 0.0001, Figure 4A, B). A systematic kinetic analysis comparing the clinical, histological and CatWalk findings at characteristic time point showed that the degree of inflammation, the number of osteoclasts and the observed gait changes mirrored the clinical course of PIA, showing the highest values in the acute phase, where also arthritis scores were highest (Figure 4C). Interestingly, early inflammatory infiltrates and gait changes (reduction of print area and stance phase shortening), but not osteoclasts or erosions could be observed prior to onset of clinically visible arthritis.

### Gait analysis detects changes prior to the onset of clinical symptoms of arthritis and can thus be used to precisely spot the timing of histological assessment of the earliest phase of PIA

**Figure 5:**
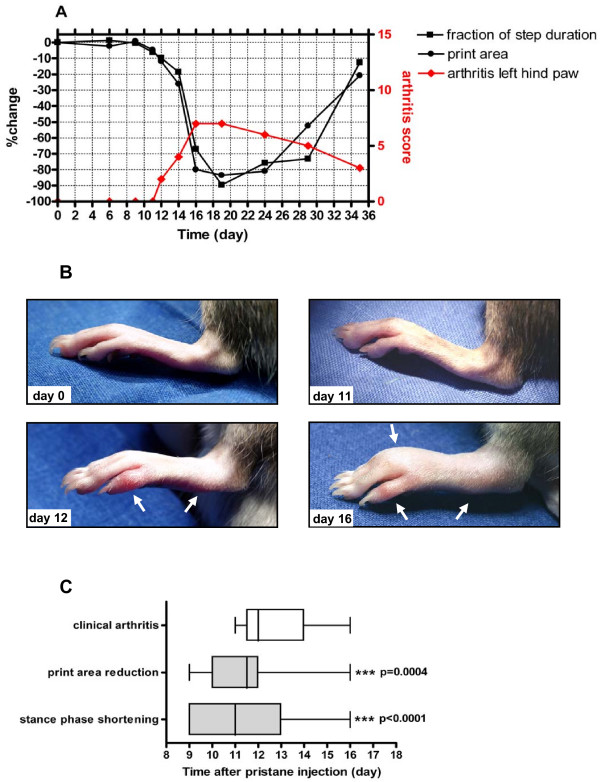
**Gait changes occur before visible arthritis is apparent**. **(A) **Time course of the print area and the duration of stance phase measured as the fraction of total step duration of a representative left hind paw after pristane-injection. **(B) **Photographic images of the left hind paw at characteristic time points (before pristane injection = Day 0, clinically unaffected joint showing gait changes = Day 11, onset of clinical PIA = Day 12, full-blown acute PIA = Day 16). Arrows show location of erythema and swelling on Day 12 and Day 16. **(C) **Comparison of day of onset of significant gait changes (defined by >5% reduction of print area or shortening of stance phase time versus pre-operative values) versus clinically apparent arthritis in paws with severe arthritis (arthritis score ≥5) reveals that print area reduction and stance phase shortening occur significantly before onset of apparent PIA. Data are presented as box plots. Lines within the boxes represent the median, boxes represent the 25^th ^and 50^th ^percentiles, and lines outside the boxes show the minimum and maximum values.

Analyses of the early changes revealed that gait changes occurred significantly earlier than the onset of clinical arthritis symptoms. Figure 5 shows the full disease course in a representative paw from injection of pristane to full-blown inflammation during the acute phase of PIA. Whereas there are detectable gait changes already at Day 11 (6.23% shortening of stance phase and 5.04% reduction of print area compared to Day 0, respectively, Figure 5A), no external clinical symptoms of arthritis can be seen yet (Figure 5B). With the beginning of externally visible arthritis at Day 12 print area and stance phase are further reduced.

On average, the first clinical symptoms became discernible 12.68 ± 0.36 days after pristane-injection, while print area reduction was seen already from 11.60 ± 0.48 days after pristane-injection (1.05 ± 0.54 days before the clinical appearance of arthritis), and the mean onset of stance phase shortening was 11.35 ± 0.49 days after pristane-injection, that is, 1.20 ± 0.71 days before the onset of clinical changes (Figure 5C). Thus, gait changes assessed by CatWalk precede the onset of clinically apparent pristane-induced arthritis.

**Figure 6:**
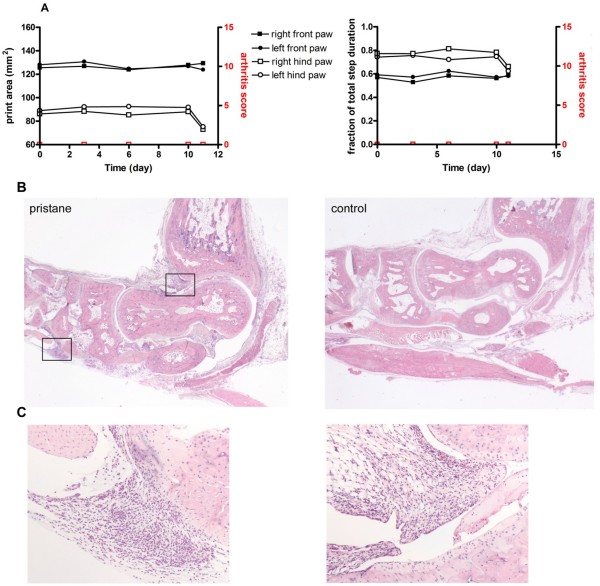
**Analysis of initial pre-clinical changes in PIA**. A female 10-week-old rat was injected with pristane and monitored by CatWalk. When gait changes suggestive of a beginning onset of PIA were detected at Day 11 (A), the rat was sacrificed. At this time no clinically visible signs of arthritis such as erythema or swelling were noticed. **(B) **Hematoxylin and eosin-stained paw sections of the pristane-injected rat's hind paws and of a PBS-injected control rat were assessed for signs of inflammation. The very early pristane-induced arthritis is characterized by synovitis and inflammation of the tendon sheaths. Boxes in (B) are shown at 10 × magnification in **(C)**.

To determine the nature of the initial events occurring in the joint shortly before the onset of clinically apparent arthritis, three pristane-injected rats were analysed by CatWalk and sacrificed when conspicuous changes in gait variables were detected (Figure 6A). Histological analysis revealed that at the time of beginning gait changes signs of inflammation were already discernible in the joint. Inflammatory infiltrates could be detected at both the tendon sheaths and the synovium (Figure 6B, C). However, osteoclasts as detectable by TRAP staining were not yet seen and toluidine blue staining hardly revealed any proteoglycan loss of the articular cartilage at this early stage of disease (not shown).

## Discussion

Recognition of early arthritis is one of the most important aspects in rheumatology, since it allows interference with the disease process before massive damage has occurred. On the other hand, information on the character of the initial pathologic events is limited, since the affected structures are not directly accessible in patients with very early disease and imaging techniques such as magnetic resonance imaging or sonography do not provide insights into the cellular processes involved. Therefore, animal models are very useful to dissect the very early arthritic processes in the joint. Pristane-induced arthritis (PIA) in the rat very closely mimics human RA since it fulfils many of its clinical criteria. However, PIA and other inducible animal models of arthritis have a rapid disease onset and progress quickly, making the dissection of initial phases of arthritis difficult. Indeed, once signs and symptoms of arthritis become discernible, not only full blown inflammation but also destructive events are likely to have already occurred [16,17]. This has been analysed by performing serial histological examinations in animals that will develop arthritis before the onset of the symptoms. However, it is difficult to envisage performing serial pre-emptive analyses in a consistent manner and, therefore, other means have to be sought. Thermal imaging was able to detect inflammatory changes already prior to onset of joint swelling in murine collagen-induced arthritis but not in rat PIA [13,14]. In the present study we have shown that the onset of inflammation in PIA is mirrored by changes in the animals' gait behaviour as detected by semi-automated locomotor analysis several days before clinically discernible signs and symptoms are apparent.

In a recent study of adjuvant-induced arthritis the experiments were terminated before the arthritic condition had become advanced [10]. The current study also provides insight into processes of remission of arthritis and their consequences for locomotion. Arthritis caused by injection of pristane caused a change in several variables typical of the way rats walk, indicating an unwillingness or inability to use the affected paws. The area touching the floor of the affected paws decreased markedly, and so did the time they rested on the floor. This was apparent about 11 to 12 days after pristane-injection, with maximum effects observed around Days 20 to 24 after pristane-injection and then gradually returning towards control values. Depending on their type, abnormalities as revealed by the CatWalk system in heavily arthritic paws were seen 0.9 to 4.3 days before clinically discernible signs of arthritis. Importantly, however, histological examination revealed that already at that time and before onset of clinically apparent arthritis significant inflammatory changes occurred in the synovial membrane, supporting previous notions on an inflammatory pre-clinical phase of arthritis [18,19].

Interestingly, in line with observations in the pre-arthritic phase of Tumor necrosis factor (TNF) α transgenic mice [16], in pre-clinical PIA there was not only inflammation of the synovial membrane, but particularly prominent inflammation of the tendon sheaths. Thus, these data suggest that periarticular tendonitis is perhaps the earliest event occurring in this model of RA.

It is assumed that the behavioural changes detected by the CatWalk system in the very early phase of arthritis are due to nociception, that is, detection of noxious stimuli and the subsequent transmission of encoded information to the brain [20] rather than due to physical impairment caused by swelling and destructive events within the joint. Pain is a perceptual process that arises in response to nociception. Although rats may not process nociceptive stimuli in the same way humans do, information gained from analysis using the CatWalk system suggests that PIA in the rat can be used to model pain in humans with inflammatory joint disease. Our data suggest that inflammation-induced noxious stimuli arise earlier than clinically visible symptoms of arthritis. As pain on motion is a major aspect of complaint in arthritic patients [21-23] elucidation of the processes involved in the initial phases of arthritis which may differ from those in later stages is essential [24]. Therefore, measuring the early clinically relevant symptoms in arthritis models may allow searching for novel therapeutics to interfere not only with pain, but particularly with the earliest events of inflammation and subsequent joint destruction and to improve physical function and quality of life of patients with RA.

## Conclusions

Using the PIA rat model of inflammatory polyarthritis we show that gait changes in affected paws observed by the CatWalk system, such as reduction of print area and shortening of the stance phase, precede the onset of clinically discernible arthritis symptoms and mirror histopathological changes during the course of PIA. Implementation of this technique in PIA reveals a beginning tenosynovitis in animals with changes in locomotive behaviour but without externally visible arthritis symptoms. Gait analysis can thus be used to pinpoint the exact timing for histological assessment of the initial inflammatory changes in animal models of inflammatory arthritis and also allows for comparing disease course and gait changes in different models in an objective manner.

## Abbreviations

a.u: arbitrary units; AUC, DA: dark agouti; EDTA: ethylenediaminetetraacetic acid; H&E: hematoxylin & eosin; NSSP: Normal Step Sequence Patterns; PBS: phosphate-buffered saline; PIA: pristane-induced arthritis; RA: rheumatoid arthritis; RI: regularity index; SEM: standard error of the mean; TNF: tumor necrosis factor; TRAP: tartrate-resistant acid phosphatase.

## Competing interests

The authors declare that they have no competing interests.

## Authors' contributions

MHH participated in the design of the study, in acquisition and analysis of data, and drafted the manuscript. RH and BN helped with data collection. HR helped to edit the manuscript. JS and GS were involved in the design of the study and helped with interpretation of data and manuscript preparation. All authors read and approved the final manuscript.
